# Developing and Testing a High-Fidelity Simulation Scenario for an Uncommon Life-Threatening Disease: Severe Malaria

**DOI:** 10.1155/2011/310524

**Published:** 2011-05-16

**Authors:** Andrew Kestler, Mary Kestler, Ravi Morchi, Steven Lowenstein, Britney Anderson

**Affiliations:** Department of Emergency Medicine, University of Colorado School of Medicine, 12401 E. 17th Avenue, B215, Aurora, CO 80045, USA

## Abstract

*Background*. Severe malaria is prevalent globally, yet it is an uncommon disease posing a challenge to education in nonendemic countries. High-fidelity simulation (sim) may be well suited to teaching its management. *Objective*. To develop and evaluate a teaching tool for severe malaria, using sim. *Methods*. A severe malaria sim scenario was developed based on 5 learning objectives. Sim sessions, conducted at an academic center, utilized METI ECS mannequin. After sim, participants received standardized debriefing and completed a test assessing learning and a survey assessing views on sim efficacy. *Results*. 29 participants included 3rd year medical students (65%), 3rd year EM residents (28%), and EM nurses (7%). Participants scored average 85% on questions related to learning objectives. 93% felt that sim was effective or very effective in teaching severe malaria, and 83% rated it most effective. All respondents felt that sim increased their knowledge on malaria. *Conclusion*. Sim is an effective tool for teaching severe malaria in and may be superior to other modalities.

## 1. Introduction

Malaria accounts for over 1 million deaths per year. The greatest burden of malaria morbidity and mortality occurs in children under 5 years of age in Africa [1]. Travel and migration increasingly transport malaria to the USA and other industrialized countries, up to 30,000 cases per year by some estimates [2]. In 2005, surveillance from the Centers for Disease Control identified 1528 cases of malaria in the USA, a 15% increase from the previous year and the greatest number since 1980 [3]. Plasmodium falciparum, the species responsible for severe malaria, accounted for 48% of the cases and caused all 7 fatalities [3]. Severe malaria refers to malaria with signs of end organ dysfunction, as manifested by coma, pulmonary edema, renal failure, circulatory collapse, or severe anemia [4]. In general, 5% of imported cases progress to severe malaria, which in turn carries 20% mortality [2]. 

In non-endemic countries, malaria poses a significant challenge to medical education. Despite its worldwide importance, malaria is uncommonly encountered by medical trainees and physicians in practice. It is a complex and lethal condition with the potential for rapid deterioration from nonspecific fever to multiorgan failure. Managing malaria thus requires time-critical actions, often on the basis of a presumptive diagnosis [4]. Unfortunately, delays in diagnosis are common [3, 5]. One can also suspect that discontinuation of quinidine as an emergency antiarrhythmic drug further reduces prescribing experience with the only widely available treatment for severe malaria in the USA. Lack of physician familiarity with the diagnosis and treatment of malaria contributes heavily to preventable malaria deaths in non-endemic countries [3, 5–7]. 

This paper proposes that the challenge of teaching severe malaria in non-endemic areas is particularly amenable to the use of high-fidelity simulation. The objective was to develop a novel teaching tool for severe malaria, using high-fidelity simulation that emphasizes predefined learning objectives for the disease. High-fidelity simulation refers to simulation scenarios with mannequins that can reproduce a wide range of patient characteristics. According to a recent review of simulation in emergency medicine [8], the “high fidelity” depends on (1) the realism of the mannequin and its software (i.e., the simulator), (2) the realism of the environment, and (3) a psychological realism as perceived by the participant.

## 2. Methods

The simulation center is equipped with the METI Emergency Care Simulator (METI ECS), a high-fidelity patient simulator that blinks, breathes, has palpable pulses, and speaks through a speaker linked to an instructor in the control room. (Additional information is available from Medical Education Technologies, Inc, Sarasota, Fl, http://www.meti.com/.) The METI ECS is located in a simulated emergency department resuscitation room complete with monitors, a code cart, and standard intravenous equipment and medications. The software of METI ECS permits the creation of multiple physiologic states, which can be programmed to worsen with time and to respond to therapeutic maneuvers. Certain physical findings that are not easily simulated on a mannequin, such as dry mucous membranes, can be communicated as observations from the patient's nurse, who is one of the simulation instructors. The simulation is directed by a lead instructor in the control room and facilitated by an in-scenario, role-playing nurse.

The authors observed several simulation scenarios in progress to learn the capabilities of the simulation center and to adapt a malaria case scenario to the simulation environment. Learning objectives were derived from a selection of recent publications searched for in MEDLINE under malaria and severe malaria, with limits “review”, and from their respective reference lists [2, 4, 9–15], as well as from the authors' prior experience in teaching malaria. Special emphasis was placed on developing learning objectives that would address typical pitfalls in the diagnosis and management of malaria in non-endemic countries. A simulation scenario was designed and pilot-tested to address the learning objectives below. 

## 3. Learning Objectives for Severe Malaria

The summarized learning objectives were as follows (see the online emergency medicine simulation scenario library for full learning objectives at http://www.emedu.org/simlibrary or at http://www.aamc.org/mededportal [16]).

**Objective 1: Recognize High-Risk Groups for Malaria**

Any traveler to an endemic area in the year prior to presentation should be considered at risk for the disease [6]. Immigrants originally from endemic countries are at particularly high risk, because they are much less likely to take malaria prophylaxis when they travel to visit family abroad [17]. The travel history is crucial to identify the susceptible patient, as any travel to the tropics constitutes a risk.

**Objective 2: Establish a Definitive or Presumptive Diagnosis of Malaria**

Fever is the most common initial manifestation of malaria, accompanied variably by headache, myalgias, jaundice, vomiting, abdominal pain, cough, and diarrhea [4]. The non-specific nature of symptoms in early, uncomplicated malaria necessitates a focused differential diagnosis that includes other life-threatening infectious diseases that benefit from prompt antibiotic treatment. Thick and thin blood smears are the backbone of laboratory diagnosis. Blood smears, however, are not always immediately read by trained personnel, and false-negatives occur. A presumptive diagnosis of malaria is warranted in any seriously ill patient who is at risk for malaria and treatment should never be delayed in the wait for a positive blood smear [4]. This frequent reliance on a presumptive scenario further highlights the importance of obtaining recent travel history on all patients presenting with fevers: unless health care workers habitually ask febrile patients about travel to the tropics, they will miss an opportunity to diagnose and treat malaria and other life-threatening tropical diseases. Additionally, some institutions may have Rapid Diagnostic Tests (RDTs) for diagnosing malaria. A Rapid Diagnostic Test (RDT) is an alternate way of quickly establishing the diagnosis of malaria infection by detecting specific P. falciparum antigens in a person's blood. RDTs have recently become available in the United States. These RDTs, whether negative or positive, must always be followed up by microscopy, as they will miss nonfalciparum malaria, and cannot measure levels of parasetemia.

**Objective 3: Identify and Treat the Complications of Severe Malaria**

Antimalarial therapy should be initiated on the basis of a presumptive diagnosis in any seriously ill patient who is at risk for malaria [4]. Coma and acidosis are the most common manifestations of severe malaria in adults. Renal failure, seizures, hypoglycemia, severe malaria, disseminated intravascular coagulation (DIC), and noncardiogenic pulmonary edema (ARDS) are other important complications of severe malaria. The most important step in managing the complications of severe malaria is the prompt initiation of antimalarial drugs [4]. All complications of malaria should be treated as they would for any critically ill patient, with the caveat that malaria patients are more prone to fluid overload than septic patients and thus require cautious fluid administration [12, 13].

**Objective 4: Know the Treatment for Severe Malaria and Its Complications**

Intravenous artesunate is now the World Health Organization- (WHO-) recommended first-line treatment for severe malaria [18]. Artesunate has proved itself superior to intravenous quinine in both adults and children, dropping treatment with quinine to second line [18–20]. Artesunate and other related compounds have a very good safety profile with few adverse effects: mild gastrointestinal symptoms may occur, while hypersensitivity reactions are on the order of 1 in 3000 [18]. Artesunate was approved as a new investigational drug in the United States on June 21st, 2007 (IND protocol no. 76,725, entitled Intravenous Artesunate for Treatment of Severe Malaria in the United States) and makes a new class of antimalarial medication, artemisinins, available in the United States [21]. High-quality intravenous artesunate is available only to malaria patients hospitalized in the United States who need intravenous treatment because of severe malaria disease, high levels of malaria parasites in the blood, inability to take oral medications, lack of timely access to intravenous quinidine, quinidine intolerance or contraindications, or quinidine failure. The drug can be provided to hospitals upon request and on an emergency basis by the CDC (see contact details below) through the DCD Drug Service or by one of the CDC Quarantine Stations located around the United States. In the USA, artesunate is currently stocked in airports in 9 major cities. Clinicians located in these major cities might therefore receive artesunate within minutes of calling the CDC, though delivery may take several hours in other cities or in other locations. The CDC reports an average call to infusion time of 7 hours (personal communication February 2011).

Because parenteral forms of artesunate and quinine are not always immediately available in the United States and other non-endemic countries and because timely initiation of an effective anti-malarial is crucial, quinidine gluconate may still occasionally be the drug of choice. Quinidine is a more effective anti-malarial than quinine but is more cardiotoxic and thus requires continuous electrocardiographic monitoring [6]. Since quinidine too has been in short supply in the USA [21], one might consider initiating a drug with anti-malarial activity (such as intravenous clindamycin or doxycycline) while awaiting delivery of artesunate. In cases of severe malaria with elevated parasitemia (>10% of red blood cells on a thin smear) that do not respond promptly to antimalarials, one may consider an exchange transfusion, although there is no strong clinical evidence to support its use [6, 15].

**Objective 5: Identify Resources for Assistance in the Management of Malaria**

Emergency medicine physicians and other critical care providers should know what resources are rapidly available to assist in the management of malaria. The website of the Centers for Disease Control (CDC) at http://www.cdc.gov/malaria is a resource, and a CDC malaria specialist is also available 24 hours a day for telephone consultations (770-488-7788 daytime, and 770-488-7100 after working hours and weekends) [5]. The same CDC malaria specialist can authorize and organize an emergency delivery of artesunate (http://www.cdc.gov/malaria/diagnosis_treatment/artesunate.html) [21]. Although a consult to an infectious disease expert as well as an inpatient pharmacist for dosing assistance can be of use, the CDC has the expertise and the means to provide up-to-date and rapid advice in treating these patients and is highly recommended by these authors.

## 4. Simulation Scenario and Evaluation

After the initial pilot test, the scenario was conducted 5 times between March 13 and September 4, 2008 in the simulation center described above. The simulations were run during weekly “simulation days” for medical students, nurses, and emergency medicine residents working in the emergency department. Some participants were directly involved in the simulated patient care, while others watched from a video-linked conference room. 

The scenario involves a 34-year-old female who complains of fever, vomiting, headache, and malaise within a week after visiting family in Nigeria. (For the full scenario, along with simulation case manager instructions, see the online simulation scenario library at http://www.emedu.org/simlibrary or http://www.aamc.org/mededportal). She did not take malaria prophylaxis but took all of her son's left-over erythromycin after becoming ill. She initially presents with a normal mental status but is febrile, tachycardic, tachypneic, and ill appearing. She soon seizes and becomes obtunded. Laboratory tests reveal a significant anion-gap acidosis, but the blood smear is not immediately available. An EKG will show sinus tachycardia with QT prolongation. If participants recognize severe malaria and treat with quinidine, the patient will develop torsades de pointes. In order to prevent a premature end to the scenario, the role-playing nurse who is part of the simulation center staff will cue participants as necessary to take actions that will keep the scenario moving and the patient alive.

The objective-related critical actions are as follows: to recognize that the patient is at high risk (Objective 1) and to order the appropriate tests for malaria and other diseases in the differential diagnosis (Objective 2); to identify seizure, coma, and acidosis as manifestations of severe malaria, and also to remember to look for or treat hypoglycemia (Objective 3); to provide supportive care, including fluid resuscitation, anticonvulsants, and endotracheal intubation as necessary (Objective 3); to start empiric therapy for severe malaria with intravenous artesunate if available (by calling the CDC if in the USA), quinidine, quinine, or a temporizing alternative; to recognize the complications of treatment (Objective 4); to seek outside help for malaria care, including specialist consultation. (Objective 5). 

After the scenario, participants received a standardized debriefing on the case, including discussion of the 5 learning objectives for severe malaria and their associated critical actions. Participants then took a 20-question survey, which included 8 multiple choice test questions relevant to the 5 learning objectives. 

## 5. Results

29 learners, 16 participants, and 13 observers were included in 5 simulation sessions. Trainees included 19 3rd year medical students, 8 3rd year EM residents, and 2 nurses (Table 1). There was no relationship between learner group (observer or participant) and trainee position (*P* = .33). Among all learners (observers and participants), simulation was rated a “very effective” instructional method by 66% (95% CI 48, 83), equivalent to actual patient care by 67% (95% CI 36, 97), and higher than problem-based learning by 6% (95% CI 0, 18) and lecture by 3% (95% CI 0, 10). 67% of participants and 62% of observers rated simulation “very effective” (*P* = .78). Postsimulation knowledge scores were equal in participants and observers (87% in both). There were also no differences in effectiveness ratings or test scores among the three trainee groups (Tables 2 and 3). 

## 6. Discussion

Our findings indicate a high learner satisfaction rate with high-fidelity simulation as a teaching tool for severe malaria. The majority of the participants in our study rated the simulation to be as effective or superior to other teaching methods. Additionally, our results suggest that not only is simulation an effective method of teaching in this case but that it was an equally effective learning tool for both observers and participants. These findings are important as they indicate that we can effectively utilize this expensive technology to educate all learners involved in the process, even if they are “just watching.” This allows us to increase the number of participants or observers in one simulation setting knowing they are being effectively educated, thus maximizing our educational investment. 

Clinical simulations, including high-fidelity simulations, are increasingly used as an assessment tool in medical education to test knowledge and competence [22]. High-fidelity simulation has often been used in teaching the management of acute situations, particularly in emergency medicine, critical care, and anesthesia. One literature review of the use of high-fidelity simulation in emergency medicine found 11 papers directly relevant to the teaching of emergency medicine [23]. Simulation applications included airway management, trauma management (ATLS), team performance training, presentation of ethical dilemmas, management of disaster/crisis scenarios, and management of medical errors. A survey of centers using simulation in anesthesia training investigated the specific reasons for the use of simulation [24]: rare events (75%) and crisis management (80%) constituted the top two reasons for use at the postgraduate level. Another study, also out of the anesthesia literature, found that simulation-based training is superior to problem-based learning in acute care scenarios [25]. 

It is likely that physicians in training in industrialized countries will encounter malaria during their training or later in their career, whether at home or abroad. Global health is an area of significant student and faculty interest and activity. The February 2008 issue of Academic Medicine is entirely devoted to the development of global health programs at academic medical centers. According to the results of an AAMC survey, 26% of medical students graduating in 2007 had had a global health experience during medical school, and 43% felt their global health training to be inadequate [26]. International rotations are now an important component of many post-graduate training programs in multiple specialties [27–29] and have an impact on recruitment [30, 31]. It is therefore absolutely relevant to teach malaria in academic centers in non-endemic countries. 

Each year, patients in industrialized countries with advanced medical care die preventable deaths from malaria, attributable to physicians' lack of familiarity with malaria and its treatment [3, 5–7]. It is therefore critical to teach the management of severe malaria effectively. How can medical educators better prepare trainees to recognize and manage malaria? High-fidelity simulation is a teaching tool well suited to the acuity and time-dependent management of severe malaria. This application has not been described in the medical literature. The rarity of malaria in non-endemic countries also supports the use of simulation, since bedside teaching is not usually feasible. In this manner, high-fidelity simulation for malaria is similar to its application in bioterrorism preparedness, another rare and life-threatening situation [32, 33]. One can easily imagine the use of simulation in teaching the management of other infectious diseases and intoxications that may be rare but provide little margin for error in diagnosis and treatment. 

## 7. Limitations

This paper does not address the long-term effectiveness of the teaching intervention, that is, its impact on the retention of knowledge and skills that have real-world applicability in the treatment of severe malaria: The evaluation was given immediately after the debriefing that reemphasized the learning objectives and critical actions. The subgroup of practicing clinicians (8 emergency medicine residents) most likely to use and benefit from new knowledge in malaria was small and perhaps too small a sample size to reach conclusions applicable to this group. 

One general limitation to simulation in medical education is the lack of evidence of its impact on patient outcomes. In general, there is good evidence that simulation-based training improves performance in simulated scenarios themselves [34]. In the particular case of manual skills and procedures, simulation-based training does actually improve real-world operational performance [34]. 

## 8. Conclusions

High-fidelity simulation constitutes a novel tool for teaching severe malaria that can meet specific learning objectives by demonstrating the need for time-dependent critical actions. In non-endemic countries it may be rare to witness a case of this disease, but, given its life-threatening nature, it is imperative that our learners are aware of this disease, know how to treat it, and have experience with its presentation, whether real or simulated, so they can manage it quickly and effectively as health care providers.

## Figures and Tables

**Table 1:** Demographics.

*N* = 29			
Level of participation	Observer	Participant	
	13 (45%)	16 (55%)	
Level of training	MS3	EM PGY3	RN
	19 (65%)	8 (28%)	2 (7%)
Specialty	EM	None listed	
	9 (31%)	20 (69%)	

**Table 2:** Prior malaria teaching, prior simulation experience, and teaching effectiveness.

*N* = 29	Yes	No				
Any prior malaria teaching	27 (93%)	2 (7%)				
Exposure to malaria teaching methods and rated effectiveness	Not effective	Minimally effective	Moderately effective	Very effective	Not exposed	Missing
Lecture	2	7	19	1	0	0
Problem-based learning (PBL)	0	3	13	1	12	0
Patient contact	0	1	2	6	19	1
Actor-based simulation	0	0	1	4	23	1
Mannequin simulation (including today's simulation)	0	1	8	19	0	1
Other (textbook)	0	0	1	0	0	28
Most effective method	Lectures	PBL	Patient contact	Actor simulation	Mannequin simulation	Other
	2	3	7	0	18	0
Prior exposure to high-fidelity simulation	Acls only	Acls and prior simulation lab	Other only	No	Missing	
	6	8	2	11	1	
Superiority of simulation teaching	Strongly disagree	Disagree	Agree	Strongly agree	Unable to assess	Missing
	0	0	11*	13	3	2

*One respondent agreed on the superiority of high-fidelity mannequin-based teaching for malaria, with the caveat that direct participating rather than observation was necessary for teaching affectiveness.

**Table 3:** Postscenario test results.

*N* = 29	Correct	Incorrect	Missing	Score (% correct)
Objective 1: recognizing high-risk groups	27	1	1	93
Objective 2: diagnosis				
Diagnostic methods	20	7	2	69
Criteria for presumptive diagnosis	24	4	1	83
Objective 3: therapy				
Criteria for presumptive therapy	27	2	0	93
First-line drug in USA	27	2	0	93
Complications of therapy	29	0	0	100
Objective 4: complications of severe malaria	16	12	1	55
Objective 5: resources for malaria	27	2	0	93

Totals	197	30	5	85
